# Barriers to Conducting Medical Research in a Newly Established Private Medical College: A Cross-Sectional Study

**DOI:** 10.7759/cureus.103812

**Published:** 2026-02-17

**Authors:** M Hasan Rajab

**Affiliations:** 1 Epidemiology and Public Health, College of Medicine, Alfaisal University, Riyadh, SAU

**Keywords:** alfaisal university, barriers to medical research, kingdom of saudi arabia, medical students, newly established university

## Abstract

Background

Medical research in emerging academic settings is an essential and rewarding endeavor that drives advancement in newly established universities within the medical education community. However, numerous barriers may impede the progress of academic medical research, particularly in developing academic environments. Identifying and understanding these barriers is critical to formulating effective strategies to overcome them.

Objective

This study aims to identify barriers to research faced by faculty members and instructors at a newly established private medical college in Saudi Arabia.

Methods

A cross-sectional study was conducted in early 2024 among approximately 120 full-time faculty members and instructors at the Alfaisal College of Medicine. Data were collected using a self-administered online questionnaire developed by the study investigators and distributed via Google Forms. A census-based sampling approach was employed. The survey was pilot-tested with three faculty members to assess clarity and feasibility. Descriptive statistics were used to summarize baseline and outcome variables. Data management and analysis were conducted using Jamovi software (version 1.2.27 for Windows). Institutional Review Board (IRB) approval was obtained before study initiation.

Results

A total of 38 faculty members and instructors responded (response rate: 32%), 63% of whom were female. Most respondents (92%) reported publishing at least one paper per year, and 79% felt encouraged by college leadership to conduct research. The most commonly reported barriers included limited funding, shortages of skilled staff, restricted access to patient populations, insufficient protected research time, challenges with IRB approval, and limited mentorship. Suggested strategies to strengthen the research culture included expanding research incentives, increasing technical support, and recruiting full-time researchers and research assistants.

Conclusions

This study identified key barriers to research at a newly established private medical college in Saudi Arabia. Despite relatively high research activity, productivity is constrained by limited funding, staffing shortages, and restricted access to research support and patient populations. Addressing these gaps through strengthened funding mechanisms, enhanced research infrastructure, expanded incentives, and dedicated research support personnel is essential for fostering a sustainable and productive research culture. Prioritizing protected research time should also be a permanent institutional goal.

## Introduction

Research is highly valued by faculty members at most academic institutions [1]. Academic institutions worldwide are ranked according to their research, innovation, and knowledge outputs [2]. Academic rankings are the manifestation of the globalization of higher education [3]. However, rankings have prompted academic institutions to skew research agendas to increase research productivity and efficiency to better the position of academic institutions in the World University Rankings. This is particularly important, especially during economic difficulties, when there might be a stronger tendency to measure outputs to ensure value for money [3].

There has been a concerning decline in the number of physician-researchers [4]. Consequently, recent studies have called for more effective strategies to integrate research into medical education curricula (knowledge and theory) and clinical training (skills and application) [4]. This decline is particularly troubling at a time when innovation in medicine is increasingly critical, given population aging and the rapid global spread of communicable diseases. Factors contributing to the decline include reduced availability of mentors and mentorship exposure, insufficient protected time for research, and limited funding opportunities [4]. In addition, most academic institutions do not permit medical students to serve as principal investigators (PIs), instead requiring faculty members to administer all student-involved research projects, which may further constrain research capacity [5].

A different study has highlighted several obstacles to medical research within academic institutions, which involve time constraints, heavy workloads, limited funding, and a shortage of motivating incentives for research [6]. One of the most challenging barriers to medical research in academia is the lack of adequate funding [6]. Academic researchers often struggle to secure the resources to support their studies. Furthermore, medical research is subject to ethical and regulatory guidelines, which can be complex and time-consuming to navigate [6]. The reported barriers to conducting and publishing research among nursing faculty members at Shaqra University, Saudi Arabia, included a lack of time due to a high teaching load and a lack of financial support [7].

In a study conducted at a public health sciences university in the United States, barriers to research were investigated among academic faculty from the fields of Dentistry, Nursing, and Allied Health Professions [8]. The study revealed that among the various obstacles to research examined, faculty overwhelmingly pointed to a shortage of time as the primary barrier to conducting research.

A recent study conducted at Alfaisal University in Saudi Arabia reported that one of the challenges in conducting research at the College of Medicine was the determination of a student's eligibility to serve as the PI for studies involving human subjects [5]. The study endorsed the practice, consistent with that of Alfaisal and other universities, of not permitting medical students to act as the PI.

To diminish the impact of these barriers to academic medical research, it is vital to understand them thoroughly and prioritize them at different institutions. It is also imperative to identify the barriers to research as a means to enhance the quality of research [9]. Additionally, increasing support for research activities among faculty members is crucial for the success of academic institutions [8]. The objective of this study was to identify barriers to research faced by faculty members and instructors at a newly established private medical college in Saudi Arabia.

## Materials and methods

Located in Riyadh, Saudi Arabia, Alfaisal University is a private, nonprofit, research-based university with over 3,000 students representing six colleges and over 40 nations [9]. The College of Medicine (COM) has a Bachelor of Medicine and Bachelor of Surgery (MBBS) program and several master's degree programs [10].

To achieve the study aim, we conducted a cross-sectional study from November 15, 2023, to March 1, 2024. The study targeted full-time faculty and instructors of the COM at Alfaisal University.

Study participants were recruited using multiple modes (email invitations, social media platforms, or in person), allowing respondents to choose their preferred method of participation. The use of multiple recruitment modes may improve coverage and increase response rates, particularly in the context of declining participation in traditional single-mode surveys. However, mixed-mode survey designs increase methodological complexity and may introduce measurement error across modes [11,12].

A census-based sampling approach was employed. The total number of faculty members and instructors at the COM was 120. Exclusion criteria included retired faculty, individuals who had left the college for any reason, and those who participated in the pilot testing of the study questionnaire.

Data were collected using a structured, self-administered online questionnaire developed in Google Forms. The questionnaire consisted of 14 items, which were carefully selected and, where applicable, adapted from existing instruments. Survey development was informed by a review of relevant literature and expert input. Face and content validity were assessed through expert review prior to survey dissemination. The questionnaire included both closed-ended and open-ended questions to collect quantitative and qualitative data. Closed-ended questions captured participants’ demographic characteristics and research experience, while open-ended questions solicited recommendations and additional comments. As part of the validation process, the draft questionnaire was pilot-tested by three faculty members. Data obtained from the pilot test were used solely to refine the survey instrument and were not included in the final analysis. No formal psychometric validation was conducted beyond pilot testing.

Data were collected between November 15 and December 15, 2023. Participants were provided with a link to the online survey, which they completed at their convenience. The survey was hosted on Google Forms, which ensured data security and anonymity. Two follow-up email reminders were sent to the participants. Descriptive statistics were used to provide a summary of both participant demographics and other relevant variables. Data management and analysis were performed using Jamovi, version 1.2.27 for Windows.

This cross-sectional study was reviewed and approved by the Alfaisal University Institutional Review Board (IRB), Riyadh, Saudi Arabia (approval no. IRB20257), under an expedited review process. Approval was obtained before the initiation of any study-related activities. Participation was voluntary, and informed consent was implied by completion of the anonymous survey. No identifiable personal information was collected, and all data were handled confidentially in accordance with IRB requirements.

## Results

The survey garnered responses from 38 COM faculty members and instructors: 18 (47%) held faculty positions in the professorial rank (assistant, associate, or full professor), while 20 (53%) were instructors. The response rate approximated 32%. Among the respondents, 14 (37%) were female, and 52% held either a Master of Science (MS) degree or a Master of Public Health (MPH) degree. The baseline characteristics of the study respondents are presented in Table 1.

**Table 1:** Baseline Characteristics (n=38)

Variable	Level	Count	Percent
Gender	Males	14	37
	Females	24	63
Highest Degree	MBBS/MD	12	32
	PharmD/PhD/DrPH	6	16
	MS/MPH	20	52
Academic Rank	Professor Rank	18	47
	Instructor	20	53
Years at the Institution	1–5	12	32
	6–10	16	42
	11–15	10	26

In terms of research-related activities, almost all (97%) study participants reported conducting research, with 92% publishing at least one paper per year and 61% attending at least one conference per year. Moreover, the majority of study respondents (79%) reported being encouraged to conduct research by the COM leadership. The research-related activities of the study respondents are depicted in Table 2.

**Table 2:** Research-Related Activities (n=38)

Variable	Level	Count	Percent
Research participation	Yes	37	97
Encouraged to do research	Yes	30	79
Publications per year	None	3	8
	1–3	19	50
	>3	16	42
Conferences per year	None	15	39
	1–3	19	50
	>3	4	11

In addition, most of the study respondents (74%) reported conducting cross-sectional studies (surveys) rather than clinical trials (Table 3).

**Table 3:** Study Type of Current Research Activity (n=38)

Study Type	Count	Percent
Medical Education	27	71
Basic Science (Lab)	19	50
Public and Community Health	15	40
Clinical Research	12	32
Preclinical (Animal) Studies	7	18

The reported barriers to research at this medical college mainly included insufficient available funding, a shortage of skilled staff, limited access to patient populations, and limited protected time for research. Additional reported barriers to research included obtaining ethical approval from the IRB and a shortage of research mentors. The reported barriers to medical research of the study respondents are depicted in Table 4.

**Table 4:** Reported Barriers to Medical Research (N=38)

Variable	Count	Percent
Limited available funding	23	61
Shortage of skilled staff	22	58
Limited access to patient populations	18	47
insufficient protected time for research	14	37
Obtaining IRB approval	11	29
Shortage of research mentors	10	26

Study participants were asked to provide recommendations to enhance the research culture at the COM. The primary recommendations included enhancing and expanding incentives to encourage research conduct, publication, and presentation of findings at national and international conferences. This would aim to motivate faculty towards engaging in clinical research rather than cross-sectional studies. Additional suggestions encompassed increasing technical support for benchwork and recruiting full-time researchers, qualified research assistants, and post-doctoral fellows. The involvement of more students in research activities was also emphasized. Furthermore, improving and facilitating the IRB process to streamline it and reduce time consumption was proposed. Enhancing access to national and international patient databases, organizing more research workshops and seminars on various study methodologies (e.g., meta-analysis and systematic reviews), and expanding research space were also recommended.

## Discussion

The findings of this study highlight key barriers that may hinder the advancement of academic medical research in newly established medical colleges. Limited funding availability emerged as one of the most frequently reported barriers, consistent with prior studies conducted in Saudi Arabia and other developing academic settings [13,14]. Inadequate financial support limits faculty's ability to conduct large-scale or longitudinal studies, often confining them to small cross-sectional or descriptive studies [14]. This financial constraint is frequently compounded by a shortage of skilled research assistants and technical personnel, which limits the capacity for data collection, analysis, and publication. Such findings reinforce the importance of institutional investment in research infrastructure and mentorship programs to encourage a productive research culture [15].

Insufficient protected time for research was another substantial barrier reported by participants, echoing barriers observed across medical colleges globally [16,17]. Faculty members in emerging academic institutions often carry heavy teaching and administrative loads, leaving limited time for scholarly activities. This imbalance diminishes research productivity and motivation [14]. Moreover, restricted access to patient populations poses an additional barrier, particularly for colleges without affiliated teaching hospitals or established clinical networks. Addressing these barriers will require institutional policies, such as allocating dedicated research time, enhancing funding mechanisms, fostering collaborations with established research centers, and integrating research mentorship, to strengthen the research environment within emerging medical colleges in Saudi Arabia and beyond.

This study has several limitations that should be considered when interpreting the findings. The most significant limitation was the low response rate, which may have increased the risk of selection and non-response bias. However, all eligible participants received the initial invitation and two follow-up email reminders. The demanding workload of faculty members and instructors may have limited their availability and willingness to complete the survey. Additionally, the scope of the study did not include the emerging role of artificial intelligence (AI) in academic research. Given the increasing use of AI-driven tools in data analysis, literature review, and manuscript preparation, excluding this domain limits the study's comprehensiveness and relevance to evolving research environments [18,19]. The use of self-reported data may introduce reporting bias, as participants' perceptions and experiences could be influenced by personal attitudes or recall errors [20,21]. Finally, no formal psychometric validation was performed beyond pilot testing. This was an exploratory study conducted at a single private medical college with a relatively small sample size, which limits the generalizability of the findings to other institutions or regions. Therefore, the results should be interpreted with caution.

## Conclusions

This article identified barriers to research that faculty members and instructors at a newly established private medical college in Saudi Arabia face. Although faculty members demonstrate strong engagement in research, their productivity is hindered by persistent structural and resource-related limitations. Inadequate funding, limited research support, and constrained access to patient populations continue to restrict the scope and quality of scholarly activity.

Addressing these barriers through improved institutional investment, enhanced research infrastructure, robust mentorship, and dedicated research personnel will be critical for strengthening the college's research capacity. By implementing these measures and expanding research incentives for high-quality research and dissemination, increasing technical support, and recruiting full-time researchers, the COM can foster a more sustainable, collaborative, and impactful research environment.
